# Supplementary material for: Intestinal Epithelium-Derived Luminally Released Extracellular Vesicles in Sepsis Exhibit the Ability to Suppress TNF-α and IL-17A Expression in Mucosal Inflammation
Source: Int J Mol Sci. 2020 Nov 10;21(22):8445. doi: 10.3390/ijms21228445 (PMC7696152; doi:10.3390/ijms21228445)
Supplement: Supplementary file 1 [file ijms-21-08445-s001.pdf]

Figure S1

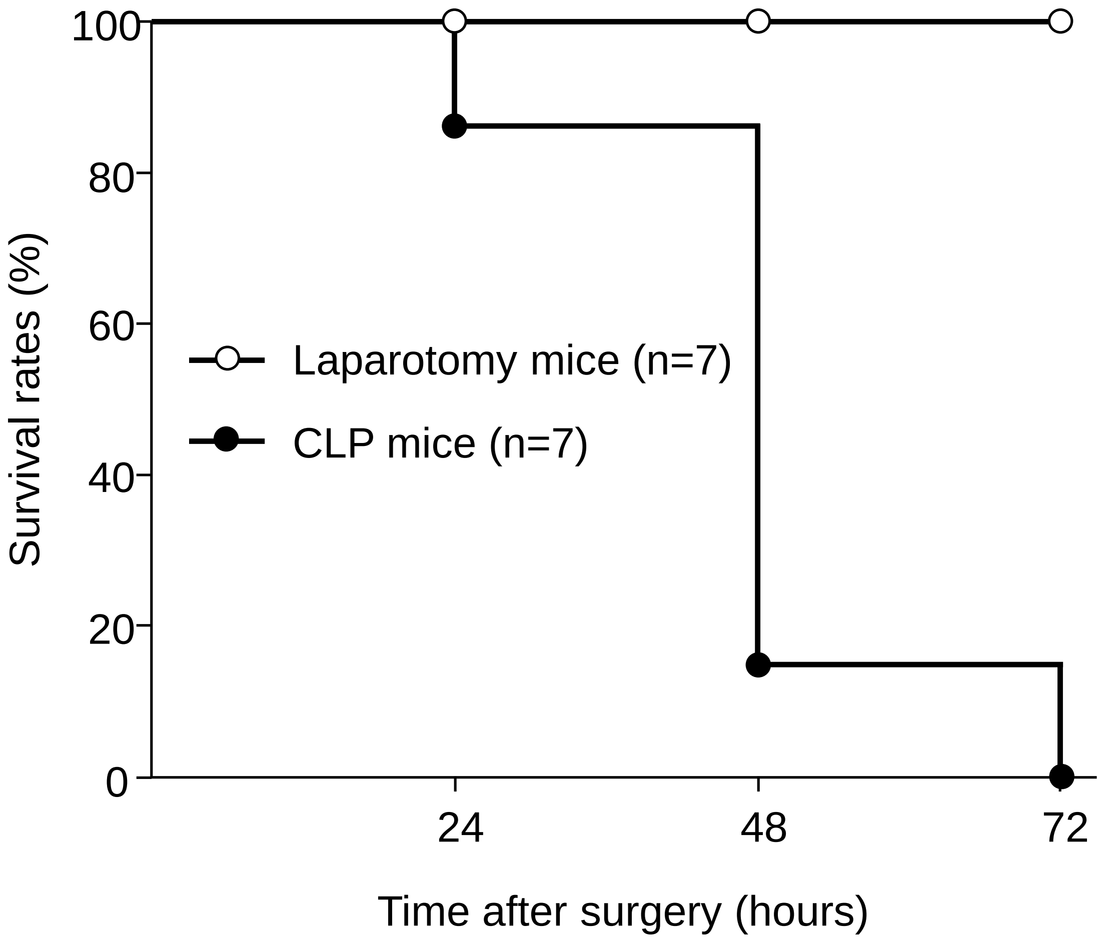

Figure S1. Monitoring of survival rates in mice following CLP or sham operation (laparotomy). Mortality of mice was observed for 72 hours. CLP (n=7) and laparotomy (n=7) mice were used in this assay.

Figure S2

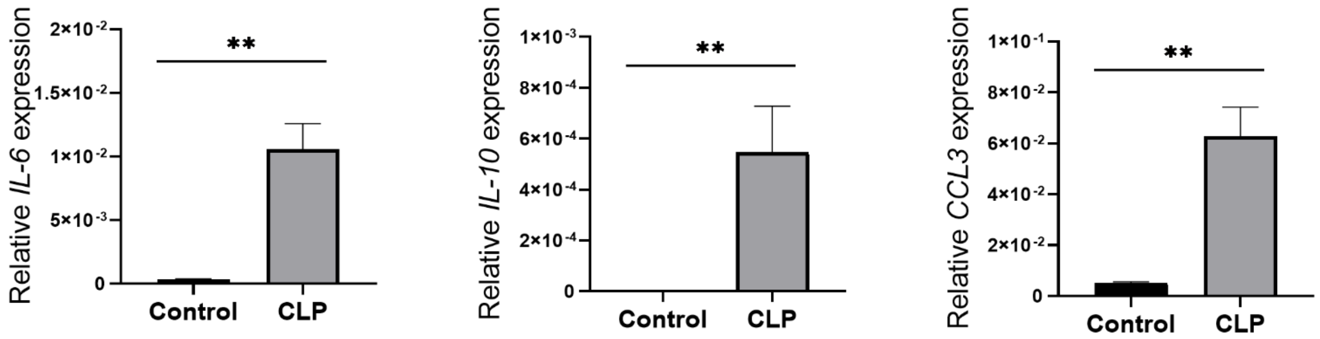

Figure S2. RT-qPCR analysis for gene expression. PBMCs were isolated from sham-operated control and CLP mice. RNA was extracted from the cells and analyzed for mRNA expression of the genes indicated. Relative expression of *IL-6, IL-10, and CCL3* to *β-actin* using $2^{-\Delta CT}$ method. Statistical significance was obtained by unpaired two-tailed t test. Whole blood from 3 mice was pooled, resulting in 4 blood samples per group (N=12). Extracted RNA was subjected to RT-qPCR assay. Results are shown as the mean ± SEM. ***p*<0.01.

Figure S3

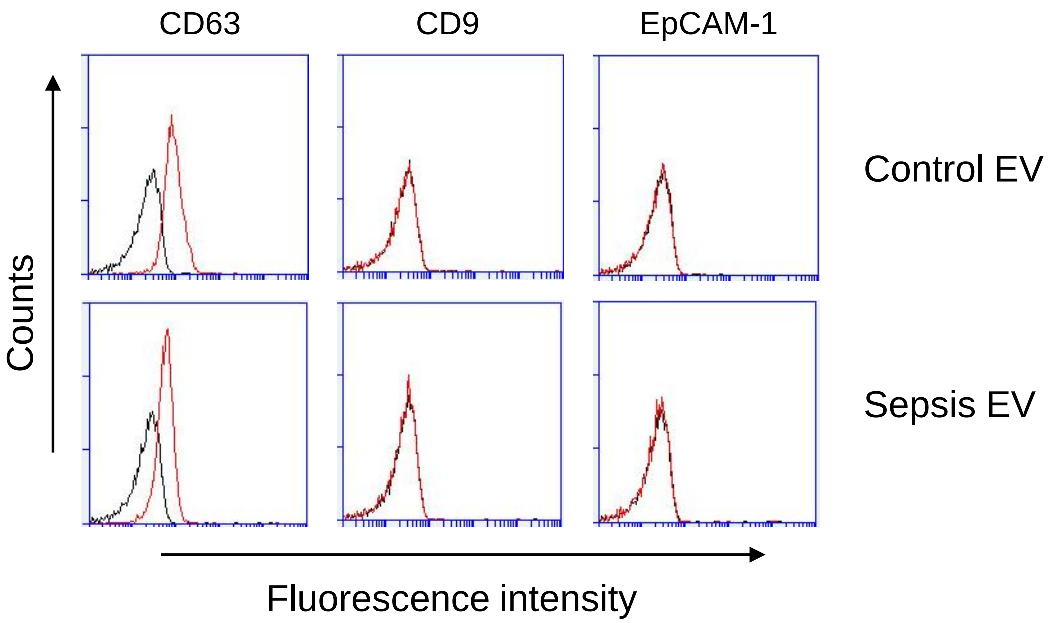

Figure S3. Flow cytometry analysis of luminal EVs isolated from control and septic mice. EVs were isolated from lavage fluids in small intestines of control and sepsis mice using differential UC. EVs were then adsorbed on 4 μm poly-L-lysine microbeads overnight. Immobilized EVs (20 μg) were stained with antibodies to mouse CD63, CD9, and EpCAM-1 and subjected to flow cytometry to evaluate their expression. Representative histograms show changes in expression of indicated markers. Black lines, isotype control; and red lines, monoclonal antibody (MAb).

Figure S4

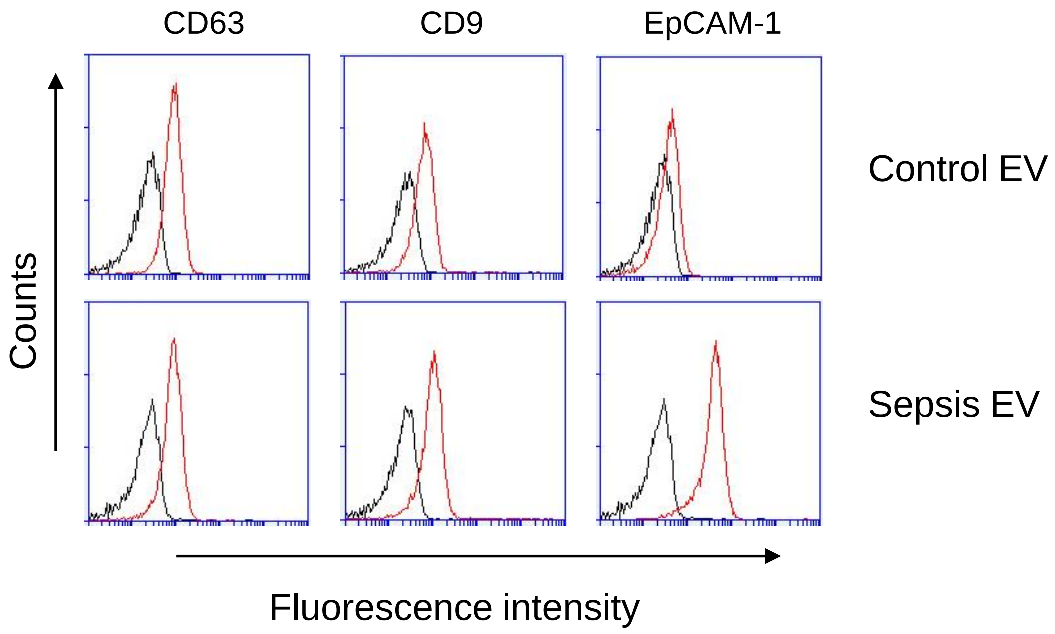

Figure S4. Flow cytometry analysis of luminal EVs isolated from large intestines of control and septic mice using C-DGUC. EVs were then adsorbed on 4 μm poly-L-lysine microbeads overnight. Immobilized EVs (20 μg) were stained with antibodies to mouse CD63, CD9, and EpCAM-1 and subjected to flow cytometry to evaluate their expression. Representative histograms show changes in the expression levels of the indicated markers. Black lines, isotype control; and red lines, monoclonal antibody (MAb).

Figure S5

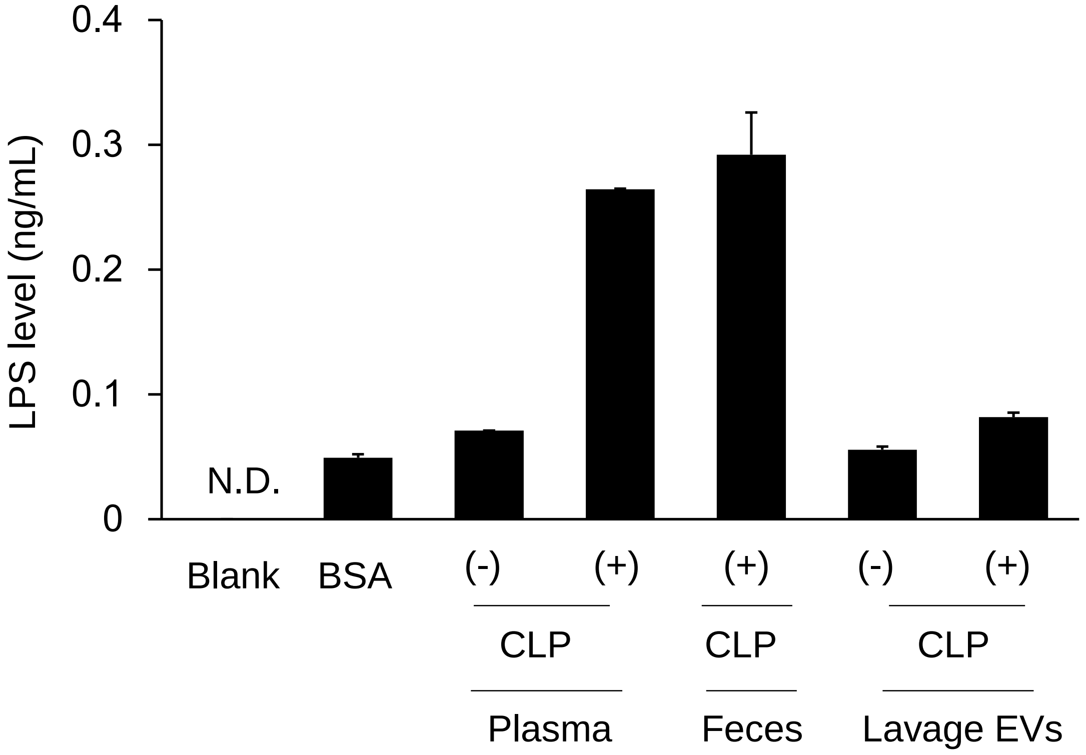

Figure S5. Determination of bacterial endotoxin (lipopolysaccharide; LPS) levels in EV samples. The EV samples were applied to an antibody-coated ELISA plate and analyzed for lipid A content. All samples had an equal concentration of 0.5 mg/ml. BSA was used as a negative control. Bar graphs represent the mean ± SEM obtained from 2-5 mice per group. BSA, bovine serum albumin.

Figure S6

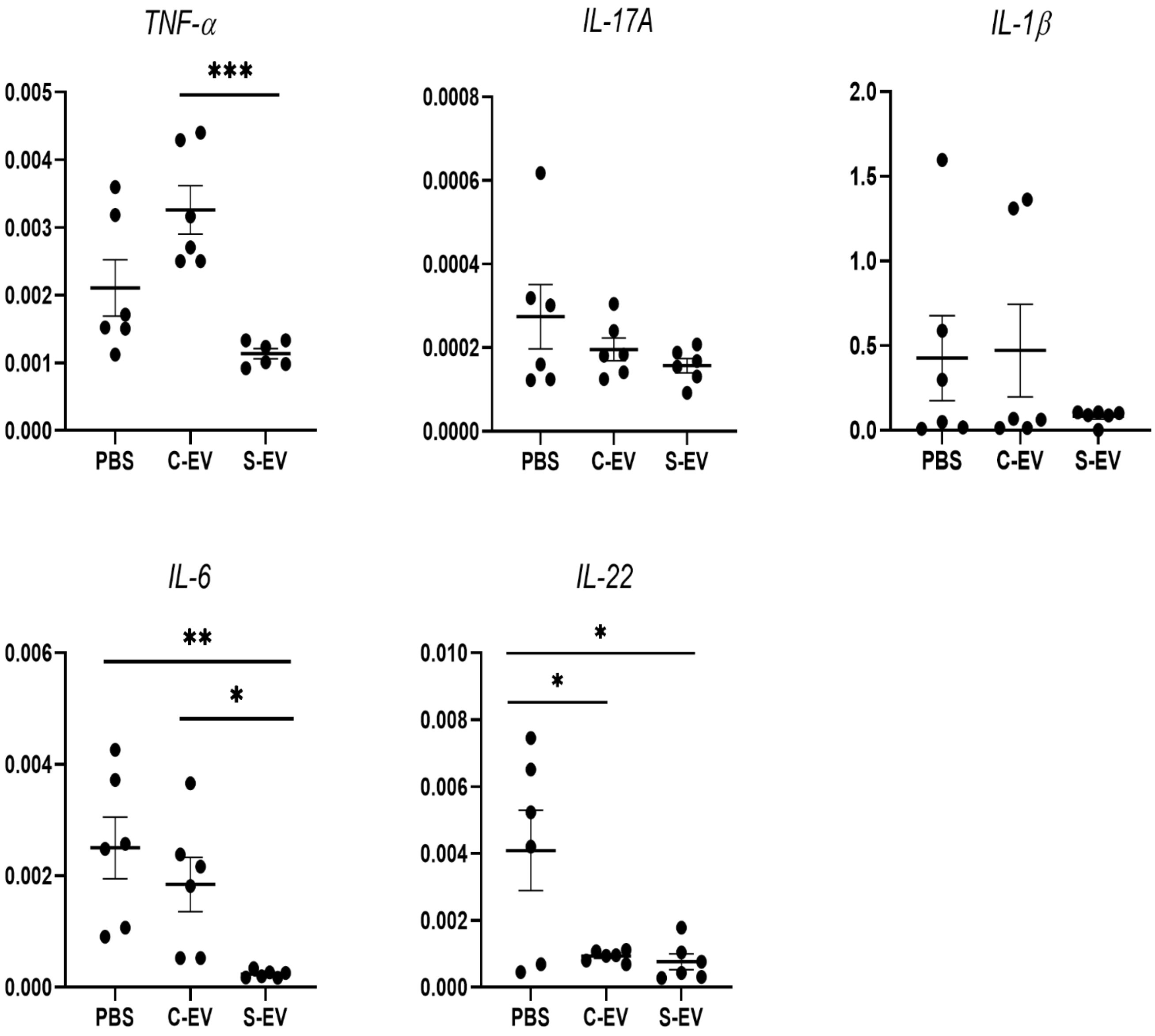

Figure S6. RT-qPCR analysis for gene expressions of *TNF-α, IL-17A, IL-1β, IL-6, and IL-22*. Relative expression of genes in ileal-loop tissues of the healthy mice treated with the EVs isolated by UC. PBS, phosphate-buffered saline (200 μL); C-EV, control EVs (50 μg/200 μL) injected; S-EV, sepsis EVs (50 μg/200 μL) injected into ileal space. Other abbreviations: TNF-α, tumor necrosis factor-α; IL, interleukin. N=6 mice per group. *p<0.05; **p<0.01; and ***p<0.001.

Figure S7

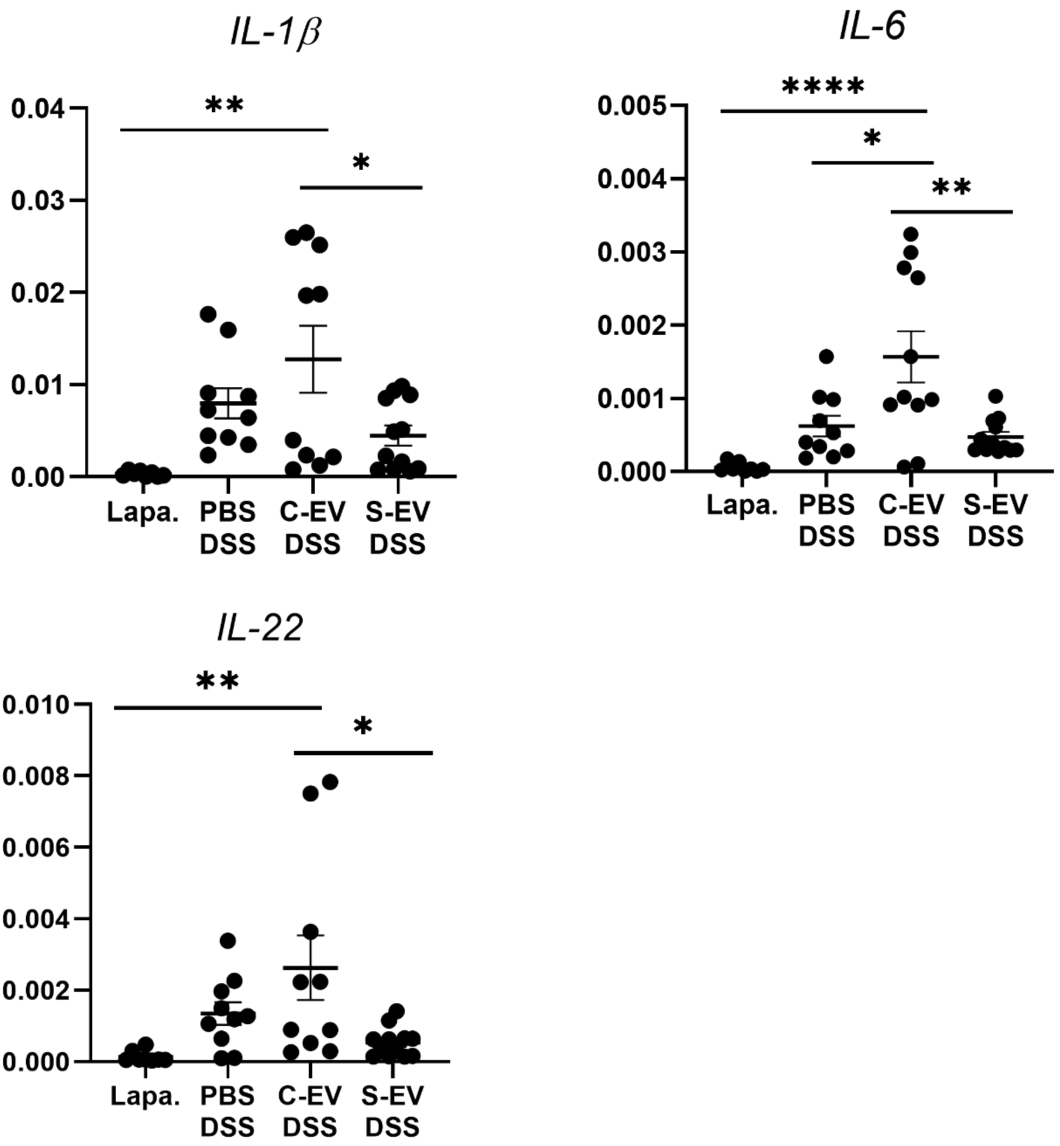

Figure S7. RT-qPCR analysis for gene expressions of *IL-1β, IL-6, and IL-22*. Relative expression of genes in ileal-loop tissues of the gut-inflamed mice treated with the EVs isolated by UC. Lapa, Laparotomy; PBS, phosphate-buffered saline (200 μL); C-EV, control EVs (50 μg/200 μL) injected; S-EV, sepsis EVs (50 μg/200 μL) injected into ileal space. Other abbreviations: TNF-α, tumor necrosis factor-α; IL, interleukin. N=8-12 mice per group. *p<0.05; **p<0.01; and ***p<0.001.

Figure S8

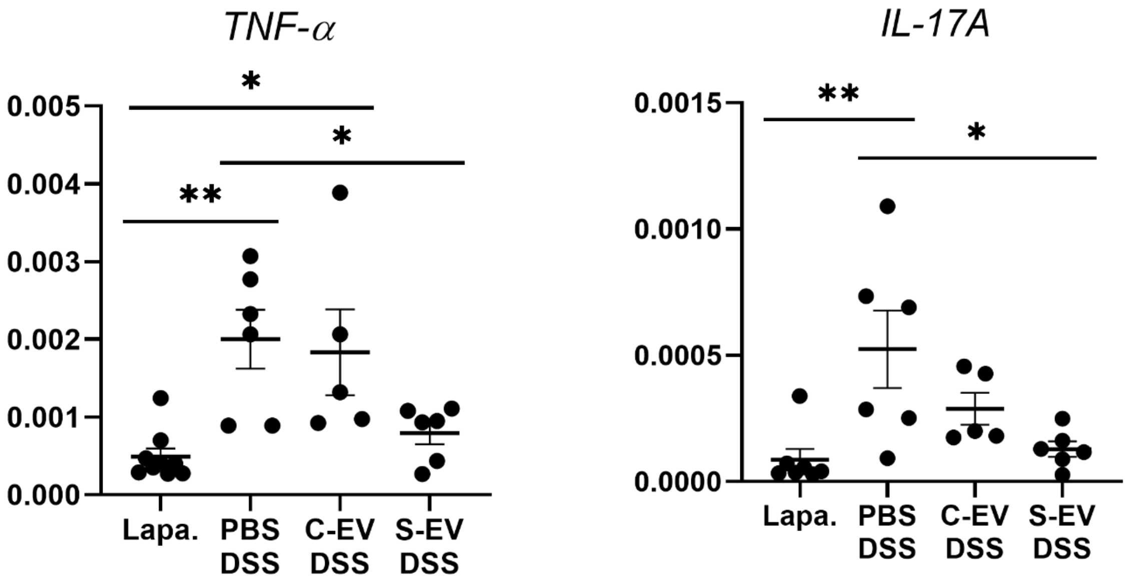

Figure S8. RT-qPCR analysis for gene expressions of *TNF-α and IL-17A*. Relative expression of genes in ileal-loop tissues of the gut-inflamed mice treated with the EVs isolated by C-DGUC. Lapa, Laparotomy; PBS, phosphate-buffered saline (200 μL); C-EV, control EVs (50 μg/200 μL) injected; S-EV, sepsis EVs (50 μg/200 μL) injected into ileal space;. Other abbreviations: TNF-α, tumor necrosis factor-α; IL-!7A, interleukin-17A. N=5-8 mice per group. *p<0.05; and **p<0.01.

Figure S9

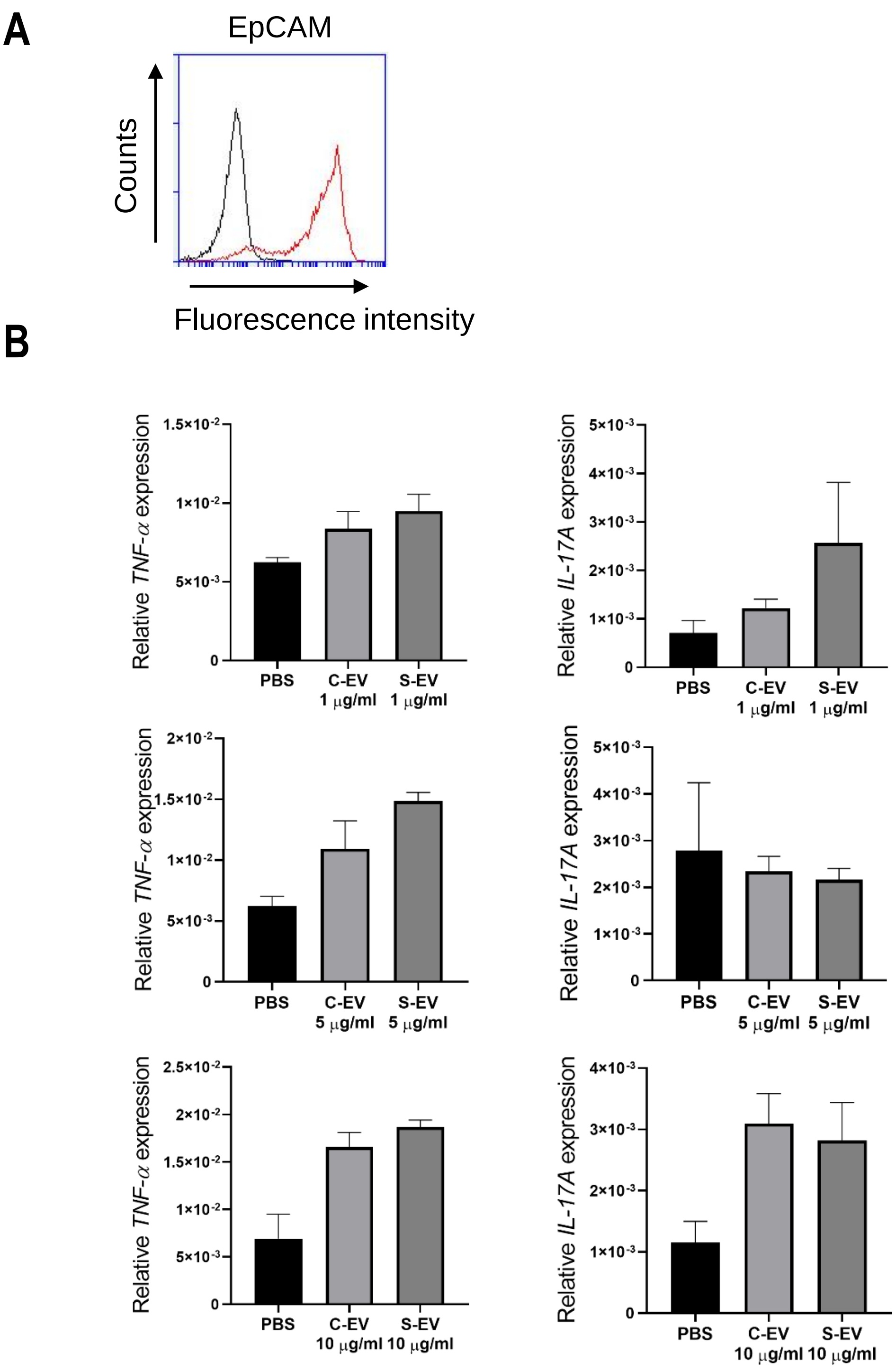

Figure S9. Confirmation of EpCAM expression and analysis of EV effects on altering *TNF-α and IL-17A* expressions in mouse IECs. (A) Representative histogram for analyzing EpCAM expression using flow cytometry. Black line, isotype control; and red line, anti-EpCAM MAb. (B) The IECs were treated with indicated doses of EVs for 24 hours and then subjected to RT-qPCR. Relative expression of both genes to *β-actin* was analyzed using $2^{-\Delta CT}$ method. 4 mice per group were used to isolate IECs and 3 to 4 independent experiments were conducted. Results are shown as the mean ± SEM.

Figure S10

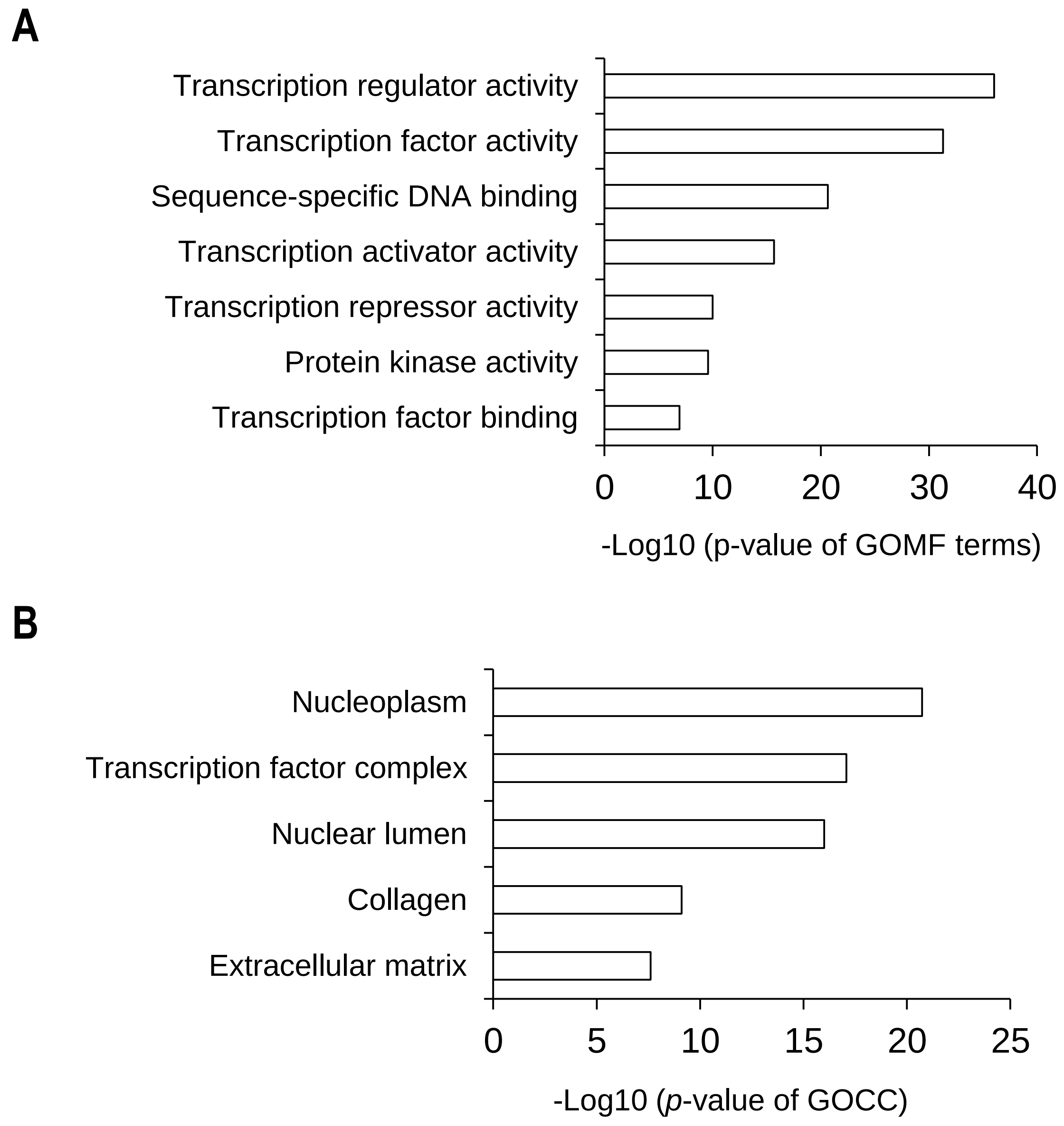

Figure S10. Gene Ontology (GO) enrichment analysis. (A) Gene Ontology Molecular Function (GOMF) terms enriched in target genes shared by upregulated microRNAs in septic EVs. (B) Gene Ontology Cellular Component (GOCC) terms enriched in target genes shared by upregulated microRNAs in septic EVs.

Table S1. Primer sequences used in RT-qPCR

	Forward (5' → 3')	Reverse (5' → 3')
TNF-α	ATAGCTCCCAGAAAAGCAAGC	CACCCCGAAGTTCAGTAGACA
IL-17A	TCTCCACCGCAATGAAGACC	CACACCCACCAGCATCTTCT
IL-1β	GCCTTGGGCCTCAAAGGAAAGAATC	GGAAGACACAGATTCCATGGTGAAG
IL-6	TGGAGTCACAGAAGGAGTGGCTAAG	TCTGACCACAGTGAGGAATGTCCAC
IL-10	ATAACTGCACCCACTTCCCA	GGGCATCACTTCTACCAGGT
IL-22	TTGAGGTGTCCAACTTCCAGCA	AGCCGGACGTCTGTGTTGTTA
CCL3	TGAAACCAGCAGCCTTTGCTC	AGGCATTCAGTTCCAGGTCAGTG
β-actin	CATCGTACTCCTGCTTGCTG	AGCGCAAGTACTCTGTGTGG
miR-19a	TGTGCAAATCTATGCAAAACTGA	Universal primer
miR-21a	TAGCTTATCAGACTGATGTTGA	Universal primer
miR-22	AAGCTGCCAGTTGAAGAACTGT	Universal primer
miR-27a	TTCACAGTGGCTAAGTTCCGC	Universal primer
miR-103-2	AGCTTCTTTACAGTGCTGCCTTG	Universal primer
miR-107	AGCAGCATTGTACAGGGCTATCA	Universal primer
miR-126a	TCGTACCGTGAGTAATAATGCG	Universal primer
miR-146b	TGAGAACTGAATTCCATAGGCT	Universal primer
miR-182	TTTGGCAATGGTAGAACTCACACCG	Universal primer
miR-200b	TAATACTGCCTGGTAATGATGA	Universal primer
miR-203	GTGAAATGTTTAGGACCACTAG	Universal primer
miR-762	GGGGCTGGGGCCGGGACAGAGC	Universal primer
U6	GCGCGTCGTGAAGCGTTC	GTGCAGGGTCCGAGGT

Table S2. All miRNAs detected, RPKM values, and fold changes

miRNA	ENSEMBL No.	RPKM		Fold change
		Control EV	Sepsis EV	(Sepsis/Control EV)
Mir6236	ENSMUSG00000098973.1	439627	606258	1.379028131
Mir320	ENSMUSG00000065528.1	3538.031	49236.06	13.91623194
Mir6240	ENSMUSG00000098343.1	25941.69	37420	1.442465776
Mir6239	ENSMUSG00000098648.1	8929.15	18945.94	2.121807787
Mir21a	ENSMUSG00000065455.1	718.601	16479.41	22.93262882
Mir194-1	ENSMUST00000083647.1	2341.455	13078.69	5.585710594
Mir192	ENSMUSG00000065523.1	1360.412	10876.74	7.995180872
Mir200a	ENSMUST00000083466.1	225.1512	9292.76	41.27341982
Mir23a	ENSMUSG00000065611.1	212.0566	7130.2	33.62404188
Mir194-2	ENSMUSG00000065582.1	852.43	6371.319	7.474301702
Mir6538	ENSMUST00000183713.1	1926.7486	4714.477	2.446856326
Mir2137	ENSMUSG00000089357.1	1657.098	4630.18	2.794149773
Mir30d	ENSMUSG00000065437.1	447.204	4496.043	10.05367349
Mir200c	ENSMUSG00000065462.2	297.7676	3704.962	12.44246184
Mir205	ENSMUST00000083599.1	178.53	3678.86	20.60639668
Mir26a-2	ENSMUSG00000065430.1	450.714	3621.92	8.03596072
Mir200b	ENSMUST00000083615.2	210.9703	3569.403	16.91898338
Mir451a	ENSMUSG00000070065.1	2618.429	3531.736	1.348799605
Mir145a	ENSMUST00000083658.1	2828.588	3512.51	1.24178919
Mir10a	ENSMUSG00000065519.1	378.0462	3413.202	9.028531433
Mir215	ENSMUSG00000065562.1	511.3293	3355.749	6.56279427
Mir26a-1	ENSMUST00000083579.1	362.995	3071.744	8.462221243
Mirlet7b	ENSMUST00000083630.1	603.601	2684.378	4.447272287
Mir30c-2	ENSMUSG00000065567.1	304.91402	2565.767	8.414722944
Mir429	ENSMUST00000083493.1	317.0862	2523.109	7.957170637
Mir141	ENSMUST00000083540.1	0	2458.856	
Mir30b	ENSMUST00000083542.2	187.2548	2307.965	12.32526483
Mir375	ENSMUST00000083682.2	12.4285	2189.302	176.151748
Mir30c-1	ENSMUSG00000065490.1	260.80818	2186.087	8.381972529
Mir139	ENSMUST00000083512.1	22.4774	2174.319	96.73356349
Mir223	ENSMUST00000102112.1	5.8594	2167.223	369.8711472
Mir143	ENSMUST00000083511.2	2896.89	2113.505	0.729577236
Mir484	ENSMUST00000093566.1	87.2046	2097.76	24.05561175
Mir30a	ENSMUSG00000065405.2	287.4851	1958.073	6.811041685
Mir151	ENSMUSG00000065612.1	108.288	1933.113	17.8515902

Mir23b	ENSMUSG00000065599.1	448.565	1752.293	3.906441653
Mir29a	ENSMUST00000083676.1	49.3168	1529.198	31.00764851
Mir5100	ENSMUSG00000092734.1	88.5256	1356.4469	15.3226513
Mirlet7g	ENSMUSG00000065440.1	260.1841	1329.393	5.109432129
Mir203	ENSMUSG00000065574.1	110.6	1312.204	11.8644123
Mirlet7c-2	ENSMUST00000083674.1	518.882	1302.325	2.509867369
Mir5126	ENSMUST00000175513.1	863.767	1298.045	1.50277216
Mir10b	ENSMUST00000083566.2	177.355	1174.837	6.624211328
Mir5112	ENSMUSG00000092638.1	35.6625	1034.777	29.01582895
Mirlet7f-2	ENSMUST00000083668.1	343.147	965.111	2.812529324
Mir27a	ENSMUST00000083510.1	193.6441	893.084	4.611986629
Mir19b-2	ENSMUST00000083539.1	112.7751	868.212	7.698614322
Mirlet7c-1	ENSMUSG00000065557.2	361.155	861.361	2.385017513
Mir140	ENSMUSG00000065439.1	17.9234	816.772	45.57014852
Mir106b	ENSMUSG00000065514.1	106.358	810.65	7.621899622
Mir19b-1	ENSMUST00000102301.1	98.448	765.517	7.775851211
Mir425	ENSMUST00000083645.1	38.0733	712.9557	18.72587089
Mir1900	ENSMUSG00000084663.1	81.2055	702.4892	8.650758877
Mir183	ENSMUSG00000065619.2	0	682.8239	
Mir103-2	ENSMUST00000083629.1	196.22	682.289	3.477163388
Mir93	ENSMUSG00000065527.1	47.9814	678.321	14.13716565
Mir103-1	ENSMUSG00000065553.1	195.8214	671.496	3.429124702
Mir182	ENSMUSG00000076361.1	40.973	669.0016	16.32786469
Mir31	ENSMUSG00000065408.1	23.6661	639.6094	27.02639641
Mir1961	ENSMUSG00000089268.1	23.7726	637.2842	26.80750949
Mir16-2	ENSMUSG00000065606.1	141.0338	628.661	4.457520112
Mir30e	ENSMUSG00000065409.1	21.6168	546.2993	25.27197828
Mir92-1	ENSMUSG00000076062.1	14.3923	537.1531	37.32225565
Mirlet7a-2	ENSMUSG00000092770.1	182.3699	521.703	2.860685892
Mirlet7a-1	ENSMUSG00000065421.2	154.9924	503.108	3.246017224
Mirlet7d	ENSMUST00000083519.1	64.3387	500.797	7.783760008
Mir150	ENSMUSG00000065495.1	0	497.815	
Mir125b-2	ENSMUSG00000065472.1	237.436	490.2327	2.064694065
Mir24-2	ENSMUST00000083607.1	140.1455	479.182	3.419175072
Mir8114	ENSMUST00000183582.1	120.96965	468.613	3.873806364
Mir29b-1	ENSMUST00000083670.1	15.4662	443.759	28.69218037
Mir17	ENSMUST00000083574.1	112.8583	431.763	3.825708876
Mir125a	ENSMUSG00000065479.1	194.38578	418.9449	2.155224009
Mir29b-2	ENSMUSG00000065412.2	12.9301	393.742	30.45158197

Mir125b-1	ENSMUSG00000093354.1	189.378	390.169	2.060265712
Mir345	ENSMUST00000083495.1	79.01686	389.5542	4.930013671
Mir26b	ENSMUST00000083534.1	260.0754	384.545	1.47859044
Mir126a	ENSMUST00000083606.1	0	373.147	
Mir3068	ENSMUST00000083004.1	101.725	365.7393	3.595372819
Mir19a	ENSMUSG00000065416.1	0	362.564	
Mir27b	ENSMUST00000083541.1	0	346.4779	
Mir652	ENSMUSG00000076011.1	65.3752	322.9616	4.940124084
Mir20a	ENSMUSG00000065442.1	0	315.9133	
Mir22	ENSMUST00000083595.1	7.16726E-30	296.117	4.13152E+31
Mir146	ENSMUSG00000065601.1	0	286.1431	
Mir15a	ENSMUST00000175266.1	0.000169239	230.509	1362032.392
Mir3060	ENSMUSG00000093080.1	101.14	229.205	2.266215147
Mir18	ENSMUST00000083469.1	78.45823	228.5649	2.913204899
Mir802	ENSMUSG00000076457.1	0	215.7689	
Mir133a-1	ENSMUST00000083465.1	0	210.5098	
Mir148a	ENSMUSG00000065505.1	8.9672	207.86	23.1800339
Mir5099	ENSMUSG00000092998.1	0	204.7156	
Mir3100	ENSMUSG00000092659.1	150.386	203.6243	1.354011012
Mir3963	ENSMUSG00000092830.1	0	197.2544	
Mir423	ENSMUSG00000065518.2	6.88462	192.9491	28.02610747
Mir1247	ENSMUSG00000080356.1	154.3272	192.5977	1.247982857
Mir107	ENSMUSG00000065594.1	54.6206	190.3094	3.484205593
Mir1945	ENSMUSG00000088544.1	22.8779	186.596	8.15616818
Mir130a	ENSMUST00000083550.1	211.524	181.3982	0.857577391
Mir5117	ENSMUSG00000065160.1	0	179.3128	
Mir574	ENSMUSG00000077042.2	121.849	175.844	1.443130432
Mir186	ENSMUST00000083497.1	22.042	171.6925	7.789333999
Mir7672	ENSMUST00000184349.1	220.219	163.1352	0.740786217
Mir185	ENSMUSG00000065464.1	0	156.223	
Mir196b	ENSMUSG00000065443.1	19.6982	153.3961	7.787315592
Mir503	ENSMUST00000102168.1	0	144.8199	
Mir744	ENSMUST00000103261.1	0	144.7817	
Mir181a-2	ENSMUST00000083489.1	0	138.6642	
Mir717	ENSMUSG00000076214.1	514.7951	138.228	0.268510714
Mir5131	ENSMUST00000175426.1	146.8719	137.7874	0.93814678
Mir1983	ENSMUST00000157523.1	16.9478	136.172	8.034789176
Mir133a-2	ENSMUST00000083526.1	0	134.5549	
Mir147	ENSMUST00000102354.2	0	134.4315	

Mir29c	ENSMUSG00000065548.1	0	131.9434	
Mirlet7i	ENSMUST00000083472.2	23.1966	128.8228	5.553520775
Mir101a	ENSMUST00000083517.1	0	115.3006	
Mir7-2	ENSMUSG00000065609.1	33.6854	113.5778	3.371721874
Mir341	ENSMUSG00000070101.1	170.6122	113.3567	0.664411455
Mir181a-1	ENSMUST00000083631.1	0	112.3415	
Mir210	ENSMUSG00000065551.1	0	110.2281	
Mir378c	ENSMUST00000184478.1	0	107.8456	
Mir365-1	ENSMUST00000083515.1	45.2313	102.5945	2.268219131
Mir199a-1	ENSMUSG00000065547.1	7.718	100.355	13.00272091
Mir762	ENSMUSG00000076454.1	37.8262	99.9531	2.642430379
Mir122	ENSMUST00000083468.1	0	99.9227	
Mir33	ENSMUST00000083531.1	0	99.5146	
Mir342	ENSMUSG00000065436.1	73.7459	97.7418	1.325386225
Mir374b	ENSMUST00000102314.1	0	97.68393	
Mir144	ENSMUSG00000065401.1	0	88.7005	
Mir99a	ENSMUSG00000065530.1	104.23	85.7015	0.822234481
Mir7-1	ENSMUSG00000065434.1	24.9955	84.7354	3.390026205
Mir718	ENSMUSG00000076127.1	78.3628	82.8833	1.057686811
Mir1957a	ENSMUSG00000088552.1	27.1997	77.7339	2.857895491
Mir25	ENSMUSG00000065394.1	0	76.1275	
Mir361	ENSMUSG00000065510.1	0	75.2315	
Mir340	ENSMUSG00000065417.1	0	74.6328	
Mir1948	ENSMUSG00000087950.1	96.2838	73.9492	0.768033667
Mir7240	ENSMUSG00000099031.1	49.0631	72.8168	1.484145926
Mir674	ENSMUST00000102421.1	0	72.1656	
Mir98	ENSMUST00000083602.2	0	67.8007	
Mir193a	ENSMUST00000083461.1	0	66.5553	
Mir196a-1	ENSMUSG00000065546.1	2.75961	66.3291	24.03567895
Mir28a	ENSMUSG00000065494.1	0	66.0922	
Mir483	ENSMUSG00000070140.1	0	64.86761	
Mir872	ENSMUST00000104728.1	0	62.6605	
Mir21c	ENSMUSG00000099326.1	0	61.093	
Mir152	ENSMUST00000083581.1	0	60.9892	
Mir700	ENSMUST00000102169.1	4.32954	59.61031	13.76827792
Mir8109	ENSMUSG00000098388.1	30.6001	58.7669	1.920480652
Mir106a	ENSMUSG00000065456.1	0	57.9363	
Mir582	ENSMUST00000103758.2	42.85608	56.198	1.311319187
Mir187	ENSMUSG00000065532.1	0	55.4029	

Mir5709	ENSMUST00000176783.1	2.85611	54.6094	19.12020195
Mir130b	ENSMUSG00000065572.1	0	53.9914	
Mir1224	ENSMUSG00000080669.1	0	53.8164	
Mir145b	ENSMUSG00000098366.1	212.8334	53.1391	0.249674628
Mir196a-2	ENSMUSG00000065488.1	0	52.774	
Mir365-2	ENSMUST00000083555.1	22.9845	52.134	2.268224238
Mir96_	ENSMUSG00000065586.1	0	51.6659	
Mir3106	ENSMUST00000175401.1	343.55	51.0791	0.14868025
Mir92-2	ENSMUST00000083679.1	0	48.07866	
Mir99b	ENSMUST00000083462.1	0	47.1088	
Mir149	ENSMUSG00000065470.1	0	46.38507	
Mir20b	ENSMUST00000102087.1	0	45.5841	
Mir128-1	ENSMUST00000083586.1	0	45.23755	
Mir7075	ENSMUST00000184187.1	36.775788	43.3489	1.17873477
Mir877	ENSMUST00000104738.1	13.4901	42.9332	3.182570922
Mir1901	ENSMUSG00000084565.1	24.8797	42.8569	1.722564983
Mir136	ENSMUSG00000070129.1	0	42.5626	
Mir5106	ENSMUST00000174936.1	0	40.8126	
Mir6958	ENSMUSG00000098663.1	0	40.2843	
Mir133b	ENSMUSG00000065480.1	0	40.02365	
Mir434	ENSMUSG00000070133.1	0	39.13195	
Mir615	ENSMUST00000102056.1	45.2722	38.88904	0.859004864
Mir153	ENSMUSG00000065538.1	0	38.6359	
Mir6936	ENSMUSG00000098282.1	42.3006	38.5199	0.910623017
Mir199b	ENSMUSG00000092807.1	9.03561	37.6519	4.16705679
Mir15b	ENSMUSG00000065580.1	7.69686	37.1318	4.824278992
Mir7008	ENSMUSG00000098890.1	145.3824	36.59128	0.251689888
Mir1839	ENSMUST00000175366.1	33.2215	35.80242	1.077688244
Mir181b-1	ENSMUST00000083524.1	0	35.6269	
Mir301	ENSMUSG00000065589.1	16.7058	35.1968	2.10686109
Mir7654	ENSMUSG00000098917.1	7.5287	35.1494	4.668721031
mmu-mir-191	ENSMUSG00000103103.1	4.88461	34.48747	7.060434712
Mir7003	ENSMUSG00000099091.1	0	33.7996	
Mir221	ENSMUSG00000065422.1	0	32.6159	
Mir100	ENSMUSG00000093011.1	0	32.3572	
Mir412	ENSMUST00000083636.1	17.6188	31.238	1.772992485
Mir6933	ENSMUST00000184982.1	0	31.2323	
Mir362	ENSMUST00000093592.1	0	30.85722	
Mir199a-2	ENSMUSG00000070126.1	1.87665	30.7005	16.3592039

Mir339	ENSMUST00000083659.1	0	30.69531	
Mir128-2	ENSMUST00000083507.1	13.8653	30.57908	2.205439478
Mir101b	ENSMUSG00000065556.1	8.22029	29.558	3.59573689
Mir455	ENSMUST00000093594.1	0	29.25225	
Mir324	ENSMUST00000083600.1	0	28.8558	
Mir672	ENSMUST00000102331.1	0	28.3244	
Mir541	ENSMUST00000102097.1	0	28.20714	
Mir8102	ENSMUSG00000098919.1	38.71212	27.91912	0.721198426
Mir326	ENSMUST00000083637.1	0	27.43728	
Mir337	ENSMUSG00000065526.3	0	26.9399	
Mir148b	ENSMUSG00000065560.1	0	26.74135	
Mir181b-2	ENSMUST00000083644.1	0	26.73158	
Mir668	ENSMUST00000183620.1	6.68318	26.12231	3.908664737
Mir677	ENSMUSG00000093245.1	0	26.06787	
Mir7662	ENSMUST00000184761.1	1.70698	25.1882	14.75600183
Mir7040	ENSMUST00000184661.1	7.61529	24.9814	3.280426615
Mir3113	ENSMUSG00000093219.1	18.321	24.93174	1.360828557
Mir34a	ENSMUST00000083559.1	58.8817	23.56345	0.400182909
Mir328	ENSMUST00000093622.1	0	23.127	
Mir675	ENSMUSG00000076275.1	0	22.7237	
Mir7071	ENSMUST00000184847.1	60.0436	22.4751	0.374312999
Mir330	ENSMUST00000083609.1	0	22.09056	
Mir214	ENSMUSG00000065516.1	0	21.9718	
Mir190a	ENSMUST00000102424.1	0	21.7084	
Mir421	ENSMUST00000083575.2	0	21.5385	
Mir146b	ENSMUSG00000070127.2	0	21.29503	
Mir92b	ENSMUST00000102300.2	0	21.1522	
Mir3473d	ENSMUST00000175346.1	0	20.9582	
Mir7648	ENSMUST00000183650.1	0	20.544	
Mir222	ENSMUST00000083537.1	0	20.18761	
Mir1932	ENSMUSG00000088015.1	22.0367	20.058	0.910208879
Mir486	ENSMUSG00000070084.3	3.78775	19.2763	5.08911623
Mir758	ENSMUST00000103260.1	0	18.84647	
Mir224	ENSMUSG00000065542.1	3.59529	18.2243	5.068937415
Mir181c	ENSMUSG00000065483.1	1.24558	17.4863	14.03868078
Mir1897	ENSMUSG00000084530.2	21.79756	16.84071	0.772596107
Mir212	ENSMUST00000083656.1	16.0107	16.57673	1.035353233
Mir490	ENSMUSG00000070075.1	0	16.5689	
Mir7235	ENSMUST00000183796.1	0	16.0079	

Mir3569	ENSMUSG00000098553.1	22.8961	15.943	0.696319461
Mir5625	ENSMUSG00000093710.1	37.8087	15.8649	0.419609772
Mir500	ENSMUSG00000070108.2	0	15.85214	
Mir7076	ENSMUSG00000099129.1	0	15.8361685	
Mir669a-11	ENSMUST00000179742.1	0	15.1306	
Mir598	ENSMUST00000102094.3	23.7658	15.0182	0.631924867
Mir6918	ENSMUSG00000098296.1	0	14.8119	
Mir501	ENSMUST00000102296.1	459.5762	14.53529	0.031627595
Mir693	ENSMUST00000102187.1	0	14.033524	
Mir1960	ENSMUSG00000088113.1	0	13.6301	
Mir3096	ENSMUST00000116685.1	0	13.586	
Mir369	ENSMUST00000083627.2	4.39962	13.3836	3.04198999
Mir3077	ENSMUSG00000092784.1	4.13509	13.25448	3.205366751
Mir379	ENSMUSG00000065498.3	0	13.024	
Mir219a-1	ENSMUSG00000065555.1	0.000166892	12.934877	77504.47595
Mir195a	ENSMUSG00000065411.2	104.2532	12.5854	0.120719556
Mir411	ENSMUSG00000065477.2	0	12.54403	
Mir7211	ENSMUST00000184071.1	0	12.4968	
Mir298	ENSMUSG00000065410.1	0	12.1373	
Mir3547	ENSMUSG00000093202.2	0	12.12809	
Mir6959	ENSMUST00000184827.1	0	12.121298	
Mir6984	ENSMUST00000185105.1	0	12.0134	
Mir532	ENSMUSG00000070139.1	0	11.5761	
Mir127	ENSMUSG00000070076.1	0	11.5635	
Mir331	ENSMUSG00000065607.1	0	11.428	
Mir8101	ENSMUSG00000098863.1	116.8116	11.426285	0.097818068
Mir671	ENSMUSG00000076387.1	11.8732	11.370882	0.957693124
Mir154	ENSMUST00000083514.1	99.4588	11.3568	0.114185974
Mir3104	ENSMUSG00000092652.1	0	11.3046	
Mir5122	ENSMUSG00000092745.1	2.12116	11.1551	5.258962077
Mir335	ENSMUST00000083567.1	0	11.12344	
Mir467d	ENSMUST00000103833.2	0	10.9618	
Mir5130	ENSMUST00000175160.1	1.75813E-36	10.901995	6.2009E+36
Mir291b	ENSMUSG00000078032.1	16.5668	10.76067	0.649532197
Mir7046	ENSMUSG00000098802.1	0	10.5443	
Mir1199	ENSMUST00000117015.1	6.1964E-102	10.3965	1.6778E+102
Mir1968	ENSMUST00000157429.1	0	10.1329	
Mir188	ENSMUSG00000065398.1	0	10.0887	
Mir378b	ENSMUSG00000092985.1	0	9.98646	

Mir7019	ENSMUST00000183998.1	3.35473	9.48575	2.827574797
Mir300	ENSMUST00000083485.2	0	9.43908	
Mir32	ENSMUSG00000065544.1	0	9.35358	
Mir8112	ENSMUSG00000098623.1	0	9.07021	
Mir6406	ENSMUSG00000099174.1	5.401	8.76736	1.623284577
Mir7013	ENSMUSG00000099033.1	0	8.6657	
Mir6921	ENSMUSG00000098547.1	0	8.42672	
Mir206	ENSMUST00000083625.1	19.8416	8.34547	0.420604689
Mir382	ENSMUSG00000065428.3	113.742	8.11414	0.071338116
Mir450b	ENSMUSG00000076050.1	0	8.1134	
Mir6979	ENSMUSG00000098706.1	0	8.09599	
Mir329	ENSMUST00000083643.1	0	7.70899	
Mir6349	ENSMUSG00000098833.1	0	7.68822	
Mir1193	ENSMUSG00000080411.1	2.64333	7.52933	2.848426038
Mir5132	ENSMUSG00000092907.1	0	7.29308	
Mir666	ENSMUSG00000076272.1	0	7.27816	
Mir350	ENSMUSG00000065573.1	0	7.14076	
Mir137	ENSMUST00000083635.1	0.000783821	7.01463	8949.275409
Mir135a-1	ENSMUSG00000065407.1	80.9461	6.89443	0.085173097
Mir467a-3	ENSMUST00000178082.1	0	6.79213	
Mir495	ENSMUSG00000070105.2	0	6.51201	
Mir6377	ENSMUSG00000098401.1	0	6.48323	
Mir1306	ENSMUST00000116802.2	0	6.22477	
Mir487b	ENSMUST00000102265.1	0	6.08505	
Mir1934	ENSMUST00000158127.1	0	5.88617	
Mir18b	ENSMUST00000103923.2	0	5.88617	
Mir467a-9	ENSMUSG00000095222.1	0	5.80197	
Mir34b	ENSMUST00000083558.1	0	5.69628	
Mir540	ENSMUSG00000072900.1	0	5.69628	
Mir378d	ENSMUSG00000098756.1	0	5.51512	
Mir6238	ENSMUSG00000099176.1	0	5.4807	
Mir1981	ENSMUSG00000088559.1	12.6001	5.44983	0.432522758
Mir7b	ENSMUST00000083557.1	0	5.37088	
Mir3102	ENSMUST00000175555.1	0	5.31024	
Mir7070	ENSMUSG00000099076.1	0	5.26132	
Mir3474	ENSMUSG00000093120.1	0	5.07227	
Mir1249	ENSMUST00000116791.1	0	5.06969	
Mir381	ENSMUST00000083632.2	0	4.71954	
Mir1964	ENSMUST00000158514.1	0	4.66675	

Mir5108	ENSMUSG00000093017.1	4.5176	4.59573	1.017294581
Mir431	ENSMUSG00000070080.1	0	4.5801	
Mir505	ENSMUST00000093573.2	0	4.57816	
Mir676	ENSMUST00000102443.1	0	4.53657	
Mir449a	ENSMUST00000083641.1	0	4.48385	
Mir297a-2	ENSMUSG00000076983.2	0	4.41161	
Mir8120	ENSMUST00000185035.1	0	4.40676	
Mir132	ENSMUST00000083603.1	0	4.26584228	
Mir129-1	ENSMUST00000083535.1	0	4.19261	
Mir5623	ENSMUST00000176631.1	0	4.14447	
Mir322	ENSMUSG00000065418.1	20.0384	4.07699	0.203458859
Mir7011	ENSMUSG00000098655.1	0	4.04498	
Mir432	ENSMUSG00000087855.1	0	3.89028	
Mir466d	ENSMUSG00000078031.1	0	3.7106	
Mir138-1	ENSMUST00000083480.1	0	3.64399	
Mir5119	ENSMUST00000175435.1	0	3.57603	
Mir669a-1	ENSMUSG00000096583.1	0	3.56708	
Mir669a-6	ENSMUST00000102064.1	0	3.56708	
Mir8097	ENSMUST00000185150.1	47.42631	3.562015	0.075106307
Mir3968	ENSMUST00000175355.1	4.97064	3.55195	0.714586049
Mir124a-3	ENSMUST00000083520.1	30.1542	3.45043	0.114426183
Mir7045	ENSMUSG00000098762.1	0.911637	3.42507	3.757054617
Mir6356	ENSMUSG00000099300.1	0	3.3688	
Mir6943	ENSMUSG00000099058.1	0	3.35298	
Mir134	ENSMUSG00000065426.2	0	3.33856	
Mir6370	ENSMUSG00000099149.1	0	3.29664	
Mir184	ENSMUST00000083662.1	4.01674	3.26896	0.813834104
Mir138-2	ENSMUSG00000065512.2	16.0033	3.25601	0.203458662
Mir8098	ENSMUSG00000099245.1	11.3642	3.19483	0.281131096
Mir7677	ENSMUST00000184226.1	5.50993	3.192237	0.579360718
Mir760	ENSMUSG00000076456.1	0	3.141688	
Mir7027	ENSMUSG00000098731.1	19.3915	3.11573	0.160675038
Mir8104	ENSMUST00000185165.1	7.91254E-07	3.04727	3851190.642
Mir6952	ENSMUSG00000098451.1	0	2.9872	
Mir338	ENSMUSG00000065600.1	0	2.90216	
Mir99ahg	ENSMUST00000182398.3	5.17424	2.883777	0.557333444
Mir5134	ENSMUSG00000093270.1	0	2.87644	
Mir142hg	ENSMUSG00000084796.1	0.238752	2.84264	11.90624581
Mir217	ENSMUSG00000065415.1	0	2.79764	

Mir1949	ENSMUSG00000088059.1	0	2.77865	
Mir680-2	ENSMUSG00000076117.1	0	2.75013	
Mir323	ENSMUST00000083683.1	99.92444	2.67074	0.026727595
Mir218-1	ENSMUSG00000065603.1	1.0671	2.66604	2.498397526
Mir3473e	ENSMUST00000183932.1	0	2.49247	
Mir218-2	ENSMUSG00000065583.1	1.51187	2.48444	1.643289436
Mir8105	ENSMUSG00000098957.1	2.98897	2.43252	0.81383219
Mir129-2	ENSMUSG00000065511.1	0	2.35977	
Mir6244	ENSMUSG00000099311.1	12.9755	2.34033	0.180365304
Mir6916	ENSMUSG00000099292.1	0	2.30376	
Mir7666	ENSMUST00000183849.1	0	2.11558	
Mir1938	ENSMUSG00000089371.1	61.9531	2.04891	0.033071953
Mir7060	ENSMUSG00000098267.1	0	1.9121	
Mir1191	ENSMUSG00000080533.1	0	1.74251	
Mir3076	ENSMUSG00000093156.1	0	1.70507	
Mir124a-2	ENSMUSG00000093073.1	16.3536	1.63152	0.099765189
Mir6417	ENSMUSG00000098726.1	5.89705	1.59543	0.270547138
Mir3109	ENSMUSG00000093042.1	0	1.48719	
Mir124-2hg	ENSMUST00000186923.1	0.00015064	1.452937	9645.094264
Mir6981	ENSMUSG00000098608.1	0	1.43695	
Mir702	ENSMUST00000102285.1	2.11236	1.31251	0.621347687
Mir299b	ENSMUSG00000093291.1	0	1.17333	
Mir6941	ENSMUST00000184711.1	2.19302	1.15342	0.525950516
Mir8111	ENSMUSG00000098823.1	0	1.04477	
Mir5110	ENSMUST00000175537.1	0	1.03513	
Mir1947	ENSMUSG00000088860.1	15.7143	0.989288	0.062954634
Mir7068	ENSMUSG00000099079.1	4.7437	0.897642	0.18922824
Mir3074-1	ENSMUSG00000092741.1	5.76691	0.782218	0.135639016
Mir22hg	ENSMUST00000134345.1	2.17207	0.51152	0.235498856
Mir124-2hg	ENSMUST00000189754.1	1.08238	0.24572323	0.227021219
Mir124-2hg	ENSMUST00000189467.1	3.70091	0.209082	0.056494754
Mir124a-1	ENSMUST00000083663.1	30.7759	0.00040736	1.32362E-05
Mir1896	ENSMUST00000122641.1	18.8082	0.00011116	5.90992E-06
Mir99ahg	ENSMUST00000182548.1	2.016994	8.8581E-07	4.39171E-07
Mir6944	ENSMUST00000182548.1	86.5956	0	0
Mir7044	ENSMUSG00000098583.1	63.6571	0	0
Mir7223	ENSMUST00000183433.1	54.9027	0	0
Mir494	ENSMUSG00000070141.2	54.2112	0	0
Mir7671	ENSMUSG00000099153.1	39.2505	0	0

Mir7007	ENSMUST00000184606.1	26.6807	0	0
Mir6368	ENSMUSG00000099158.1	19.9119	0	0
Mir878	ENSMUST00000104680.1	17.1584	0	0
Mir8092	ENSMUSG00000099118.1	16.7424	0	0
Mir707	ENSMUSG00000076051.1	15.8084	0	0
Mir1231	ENSMUST00000175639.1	15.0984	0	0
Mir6414	ENSMUSG00000098264.1	14.954	0	0
Mir292	ENSMUSG00000078041.1	14.954	0	0
Mir204	ENSMUSG00000065507.2	12.485	0	0
Mir6996	ENSMUSG00000098548.1	11.694	0	0
Mir7058	ENSMUST00000184637.1	9.9898	0	0
Mir7236	ENSMUST00000184435.1	9.1429	0	0
Mir688	ENSMUSG00000076009.1	8.86544	0	0
Mir6385	ENSMUSG00000098886.1	7.66388	0	0
Mir3062	ENSMUST00000174986.1	7.57894	0	0
Mir6937	ENSMUSG00000098579.1	5.47507	0	0
Mir1197	ENSMUST00000117037.1	5.401	0	0
Mir7023	ENSMUST00000185093.1	5.0642	0	0
Mir1905	ENSMUST00000122779.1	4.98467	0	0
Mir3086	ENSMUSG00000092813.1	4.23972	0	0
Mir7016	ENSMUSG00000098422.1	3.80327	0	0
Mir7001	ENSMUSG00000099238.1	3.68065	0	0
Mir7039	ENSMUSG00000098822.1	3.12336	0	0
Mir142hg	ENSMUST00000123700.1	2.82696	0	0
Mir433	ENSMUSG00000070072.1	2.48227	0	0
Mir207	ENSMUSG00000065452.1	2.39072	0	0
Mir6335	ENSMUSG00000098734.1	2.33053	0	0
Mir6241	ENSMUST00000184225.1	2.17795	0	0
Mir6350	ENSMUSG00000098876.1	1.91597	0	0
Mir6392	ENSMUST00000184924.1	1.46402	0	0
Mir99ahg	ENSMUST00000182986.1	1.20409	0	0

Table S3. RPKM values and fold change of miRNAs targeting TNF-α and IL-17A

miRNA	ENSEMBL No.	TNF-α			IL-17A		
		RPKM		Fold change (Sepsis/ Control EV)	RPKM		Fold change (Sepsis/ Control EV)
		Control EV	Sepsis EV		Control EV	Sepsis EV	
Mir320	ENSMUSG00000065528.1				3538.031	49236.06	13.916232
Mir6240	ENSMUSG00000098343.1				25941.69	37420	1.4424658
Mir21a	ENSMUSG00000065455.1				718.601	16479.41	22.932629
Mir200a	ENSMUST00000083466.1				225.1512	9292.76	41.27342
Mir200c	ENSMUSG00000065462.2				297.7676	3704.962	12.442462
Mir26a-2	ENSMUSG00000065430.1				450.714	3621.92	8.0359607
Mir200b	ENSMUST00000083615.2				210.9703	3569.403	16.918983
Mir429	ENSMUST00000083493.1				317.0862	2523.109	7.9571706
Mir30b	ENSMUST00000083542.2				187.2548	2307.965	12.325265
Mir203	ENSMUSG00000065574.1				110.6	1312.204	11.864412
Mir10b	ENSMUST00000083566.2				177.355	1174.837	6.6242113
Mir5112	ENSMUSG00000092638.1	35.6625	1034.777	29.015829	35.6625	1034.777	29.015829
Mir27a	ENSMUST00000083510.1	193.6441	893.084	4.6119866			
Mir140	ENSMUSG00000065439.1				17.9234	816.772	45.570149
Mir183	ENSMUSG00000065619.2				0	682.8239	
Mir103-2	ENSMUST00000083629.1				196.22	682.289	3.4771634
Mir103-1	ENSMUSG00000065553.1				195.8214	671.496	3.4291247
Mir182	ENSMUSG00000076361.1				40.973	669.0016	16.327865
Mir125a	ENSMUSG00000065479.1	194.38578	418.9449	2.155224			
Mir29b-2	ENSMUSG00000065412.2				12.9301	393.742	30.451582
Mir26b	ENSMUST00000083534.1				260.0754	384.545	1.4785904
Mir126a	ENSMUST00000083606.1				0	373.147	
Mir3068	ENSMUST00000083004.1	101.725	365.7393	3.5953728	101.725	365.7393	3.5953728
Mir19a	ENSMUSG00000065416.1	0	362.564		0	362.564	
Mir27b	ENSMUST00000083541.1	0	346.4779				
Mir22	ENSMUST00000083595.1				0	296.117	
Mir146a	ENSMUSG00000065601.1	0	286.1431				
Mir3060	ENSMUSG00000093080.1				101.14	229.205	2.2662151
Mir107	ENSMUSG00000065594.1				54.6206	190.3094	3.4842056
Mir130a	ENSMUST00000083550.1	211.524	181.3982	0.8575774			
Mir185	ENSMUSG00000065464.1	0	156.223		0	156.223	

Mir196b	ENSMUSG00000065443.1	19.6982	153.3961	7.7873156	19.6982	153.3961	7.7873156
Mir101a	ENSMUST00000083517.1	0	115.3006		0	115.3006	
Mir210	ENSMUSG00000065551.1				0	110.2281	
Mir762	ENSMUSG00000076454.1	37.8262	99.9531	2.6424304			
Mir33	ENSMUST00000083531.1	0	99.5146				
Mir99a	ENSMUSG00000065530.1	104.23	85.7015	0.8222345	104.23	85.7015	0.8222345
Mir25	ENSMUSG00000065394.1	0	76.1275		0	76.1275	
Mir674	ENSMUST00000102421.1	0	72.1656				
Mir582	ENSMUST00000103758.2	42.85608	56.198	1.3113192	42.85608	56.198	1.3113192
Mir130b	ENSMUSG00000065572.1	0	53.9914		0	53.9914	
Mir96	ENSMUSG00000065586.1				0	51.6659	
Mir3106	ENSMUST00000175401.1				343.55	51.0791	0.1486803
Mir99b	ENSMUST00000083462.1	0	47.1088		0	47.1088	
Mir149	ENSMUSG00000065470.1	0	46.38507		0	46.38507	
Mir7075	ENSMUST00000184187.1	36.775788	43.3489	1.1787348			
Mir877	ENSMUST00000104738.1				13.4901	42.9332	3.1825709
Mir133b	ENSMUSG00000065480.1	0	40.02365				
Mir6936	ENSMUSG00000098282.1	42.3006	38.5199	0.910623			
Mir199b	ENSMUSG00000092807.1	9.03561	37.6519	4.1670568			
Mir1839	ENSMUST00000175366.1	33.2215	35.80242	1.0776882	33.2215	35.80242	1.0776882
Mir301a	ENSMUSG00000065589.1	16.7058	35.1968	2.1068611			
Mir7003	ENSMUSG00000099091.1	0	33.7996				
Mir100	ENSMUSG00000093011.1	0	32.3572				
Mir412	ENSMUST00000083636.1				17.6188	31.238	1.7729925
Mir101b	ENSMUSG00000065556.1	8.22029	29.558	3.5957369	8.22029	29.558	3.5957369
Mir455	ENSMUST00000093594.1	0	29.25225				
Mir326	ENSMUST00000083637.1				0	27.43728	
Mir337	ENSMUSG00000065526.3	0	26.9399				
Mir677	ENSMUSG00000093245.1	0	26.06787				
Mir7662	ENSMUST00000184761.1				1.70698	25.1882	14.756002
Mir328	ENSMUST00000093622.1	0	23.127		0	23.127	
Mir330	ENSMUST00000083609.1				0	22.09056	
Mir421	ENSMUST00000083575.2				0	21.5385	
Mir146b	ENSMUSG00000070127.2				0	21.29503	
Mir92b	ENSMUST00000102300.2	0	21.1522		0	21.1522	
Mir7648	ENSMUST00000183650.1	0	20.544				
Mir181c	ENSMUSG00000065483.1	1.24558	17.4863	14.038681			

Mir5625	ENSMUSG00000093710.1				37.8087	15.8649	0.4196098
Mir7076	ENSMUSG00000099129.1	0	15.836168				
Mir6918	ENSMUSG00000098296.1				0	14.8119	
Mir3096	ENSMUST00000116685.1	0	13.586				
Mir411	ENSMUSG00000065477.2	0	12.54403				
Mir298	ENSMUSG00000065410.1	0	12.1373				
Mir3547	ENSMUSG00000093202.2	0	12.12809				
Mir6959	ENSMUST00000184827.1	0	12.121298				
Mir6984	ENSMUST00000185105.1				0	12.0134	
Mir154	ENSMUST00000083514.1				99.4588	11.3568	0.114186
Mir335	ENSMUST00000083567.1				0	11.12344	
Mir467d	ENSMUST00000103833.2	0	10.9618				
Mir291b	ENSMUSG00000078032.1	16.5668	10.76067	0.6495322			
Mir1199	ENSMUST00000117015.1	0	10.3965		0	10.3965	
Mir188	ENSMUSG00000065398.1	0	10.0887				
Mir300	ENSMUST00000083485.2				0	9.43908	
Mir32	ENSMUSG00000065544.1	0	9.35358		0	9.35358	
Mir450b	ENSMUSG00000076050.1	0	8.1134		0	8.1134	
Mir1193	ENSMUSG00000080411.1	2.64333	7.52933	2.848426			
Mir5132	ENSMUSG00000092907.1	0	7.29308		0	7.29308	
Mir350	ENSMUSG00000065573.1	0	7.14076				
Mir137	ENSMUST00000083635.1				0	7.01463	
Mir495	ENSMUSG00000070105.2				0	6.51201	
Mir1934	ENSMUST00000158127.1				0	5.88617	
Mir540	ENSMUSG00000072900.1	0	5.69628				
Mir1981	ENSMUSG00000088559.1				12.6001	5.44983	0.4325228
Mir3102	ENSMUST00000175555.1	0	5.31024				
Mir1249	ENSMUST00000116791.1				0	5.06969	
Mir3968	ENSMUST00000175355.1				4.97064	3.55195	0.714586
Mir6370	ENSMUSG00000099149.1	0	3.29664				
Mir184	ENSMUST00000083662.1	4.01674	3.26896	0.8138341			
Mir760	ENSMUSG00000076456.1	0	3.141688				
Mir7027	ENSMUSG00000098731.1	19.3915	3.11573	0.160675			
Mir338	ENSMUSG00000065600.1	0	2.90216				
Mir323	ENSMUST00000083683.1	99.92444	2.67074	0.0267276			
Mir3076	ENSMUSG00000093156.1				0	1.70507	
Mir3109	ENSMUSG00000093042.1	0	1.48719		0	1.48719	

Mir6981	ENSMUSG00000098608.1	0	1.43695		0	1.43695	
Mir5110	ENSMUST00000175537.1	0	1.03513				
Mir7068	ENSMUSG00000099079.1				4.7437	0.897642	0.1892282
Mir3074-1	ENSMUSG00000092741.1				5.76691	0.782218	0.135639
Mir292a	ENSMUSG00000078041.1	14.954	0	0			
Mir204	ENSMUSG00000065507.2	12.485	0	0	12.485	0	0
Mir7016	ENSMUSG00000098422.1				3.80327	0	0
Mir7058	ENSMUST00000184637.1	9.9898	0	0			
Mir7236	ENSMUST00000184435.1	9.1429	0	0			
Mir3062	ENSMUST00000174986.1	7.57894	0	0			
Mir1197	ENSMUST00000117037.1	5.401	0	0			
Mir7039	ENSMUSG00000098822.1				3.12336	0	0
